# Update of randomized controlled trials evaluating cytoreductive surgery (CRS) and hyperthermic intraperitoneal chemotherapy (HIPEC) in prevention and therapy of peritoneal metastasis: a systematic review

**DOI:** 10.1515/pp-2021-0152

**Published:** 2022-03-15

**Authors:** Barbara Noiret, Guillaume Piessen, Clarisse Eveno

**Affiliations:** Department of Digestive and Oncological Surgery, Claude Huriez University Hospital, Lille, France; UMR-S1277 - CANTHER laboratory “Cancer Heterogeneity, Plasticity and Resistance to Therapies”, Lille, France

**Keywords:** cytoreductive surgery, hyperthermic intraperitoneal chemotherapy, peritoneal metastasis, prophylactic indication, randomized controlled trial, therapeutic indication

## Abstract

**Background:**

Cytoreductive surgery (CRS) and hyperthermic intraperitoneal chemotherapy (HIPEC) is associated with favorable short- and long-term oncological outcomes in highly selected patients with peritoneal metastasis (PM). The aim of our review was to review published, recruiting or ongoing randomized controlled trials (RCTs) evaluating CRS and HIPEC vs. other strategies (systemic chemotherapy or CRS alone) and to update the studies recently described in 2016.

**Content:**

Systematic review according to PRISMA guidelines. Searches for published and ongoing trials were based, respectively, on PubMed and international clinical databases since 2016.

**Summary:**

46 trials randomized 9,063 patients: 13 in colorectal cancer (3 in therapeutic strategy and 10 in prophylactic strategy), 16 in gastric cancer (4 in therapeutic strategy and 12 in prophylactic strategy) and 17 in ovarian cancer (12 in front-line therapy and 5 in recurrence settings).

**Outlook:**

In contrast to many recruiting studies, few published studies analyzed the potential advantage of CRS and HIPEC in therapeutic and prophylactic treatment of PM. The potential effect of this combined treatment has been proven in ovarian cancer in interval surgery, but remains still debated in other situations. Promising trials are currently recruiting to provide further evidence of the effectiveness of CRS and HIPEC.

## Introduction

Peritoneal metastasis (PM) is defined as peritoneal metastases of pre-existing cancers (mostly digestive or gynecological cancers) or as primary peritoneal malignancies such as malignant peritoneal mesothelioma (MPM) or pseudomyxoma peritonei (PMP). Treatment combining cytoreductive surgery (CRS) and hyperthermic intraperitoneal chemotherapy (HIPEC) has a key role in the management of all PM in highly selected patients. Several studies have widely demonstrated the benefit survival of the combined treatment in gastric cancer [1], ovarian cancer [2], appendiceal cancer and PMP [3, 4] or MPM [4]. Nevertheless, in colorectal cancer, different studies are also in favor of CRS and HIPEC [5, 6] but the role of HIPEC is still debated. Even if CRS and HIPEC have long been regarded as an aggressive procedure, this combined treatment is performed with a controlled major morbidity (MM) and mortality [7]. Several randomized clinical trials (RCTs) were recently published or are currently ongoing evaluating the contribution of CRS and HIPEC compared to other therapeutic approaches in PM. A previous revue dedicated to RCT evaluating CRS and HIPEC in prophylactic or curative treatment of PM was published by Eveno et al. in 2016 [8]. Thirty-eight RCT trials had been identified between 1980 and 2016.

The aim of this systematic review was to update results of those trials and describe new ongoing and planned studies.

## Materials and methods

### Study design

**Figure 1:**
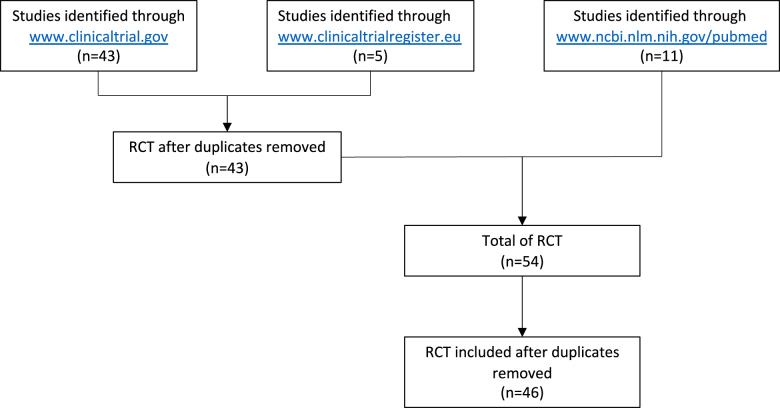
Chart flow for the study design.

This is an updated systematic review since the previous study published in 2016 [8] according to PRISMA guidelines [9]. Flow chart is detailed in Figure 1.

### Clinical trials database search strategy

We performed the same systematic search of the US National Institute of Health clinical trials database (“https://www.clinicaltrials.gov”) on November 15, 2021. The search terms were “HIPEC AND randomized AND surgery”, which identified 43 studies.

An additional search was carried out on the EC clinical trials database (https://www.clinicaltrialsregister.eu), with the search terms “surgery” AND “hyperthermic intraperitoneal chemotherapy” and “randomized”. Five studies were identified. This search was compared to the US search and five duplicates removed.

After individual review of each study protocol, RCTs were excluded according to exclusion criteria detailed in Table 1. The total number of RCTs in this study is therefore 43.

**Table 1:** Selection criteria for relevant studies.

Characteristic	Criteria
Inclusion criteria	Prospective RCT comparing the outcome(s) of CRS and HIPEC vs. systemic chemotherapy (alone or in combination). Prospective RCT comparing the outcome(s) of CRS and HIPEC vs. CRS alone.
Exclusion criteria	Comparison of various techniques of CRS and HIPEC (e.g., different pressure, time, temperature, drug, ect.). Review or metanalysis. Case-report. Other intraperitoneal chemotherapy techniques such as EPIC, PIPAC, NIPS
Patients	Patients with peritoneal carcinomatosis (PC)
Intervention	CRS and HIPEC
Outcome	Selected abstract contained information relevant to the safety and/or efficacy
Language	Only articles in English were included.

CRS, cytoreductive surgery; HIPEC, hyperthermic intraperitoneal chemotherapy; NIPS, neoadjuvant intraperitoneal systemic chemotherapy protocol; PIPAC, pressurized intraperitoneal aerosol chemotherapy; PM, peritoneal metastasis; RCT, randomized clinical trial.

### Literature database search strategy

As previously describer [8], we performed a systematic search of the US National Institute of Health literature database (“https://www.ncbi.nlm.nih.gov/pubmed”) on November 15, 2021 with the search terms “hyperthermic intraperitoneal chemotherapy” OR “continuous hyperthermic peritoneal perfusion” AND “randomized”. 11 RCT were identified and were compared to the clinical trials database.

A total of 46 studies were analyzed in this review.

## Results

Results of RCT concerning CRS and HIPEC for PM are presented by organ (colorectal, gastric and ovarian cancer).

### Colorectal cancer

Table 2 summarizes RCTs evaluating the role of CRS and HIPEC in colorectal cancer.

**Table 2:** Prospective randomized trials evaluating CRS and HIPEC in colorectal cancer.

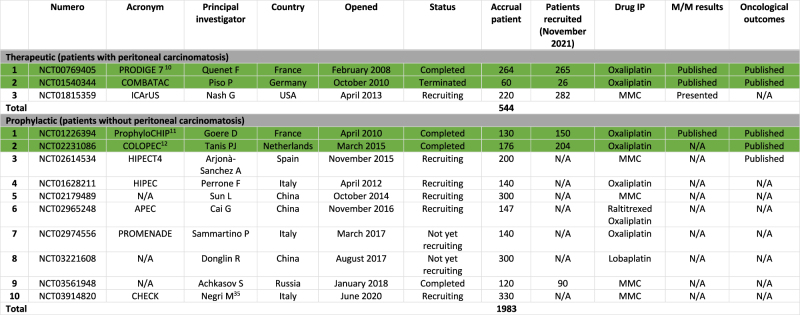

N/A, not available at date (November 2021); MMC, mitomycin. Green: results published.

#### Therapeutic indication (peritoneal metastasis)

Three RCTs have been identified in patients with PM of colorectal origin, with a planned accrual patient of 544. Two trials were closed and results have been published. One other RCT is active and still recruiting patients.

Results of the French PRODIGE 7 trial [10] were recently published in 2021. In this study, 265 patients were randomly assigned intraoperatively after CRS to receive oxaliplatin based-HIPEC or not. Patients with extraperitoneal metastases, previous HIPEC or high PCI (>25) were excluded. In both groups, complete CRS (CC-0 or CC-1) was performed. In the experimental group, oxaliplatin based-HIPEC was administered by open or closed abdominal techniques over 30 min. After a median follow-up of 63.8 months, Quenet et al. showed no difference in OS between CRS alone and CRS plus HIPEC, respectively, 41.2 and 41.7 months (p=0.99). In the same way, no evidence of a relapse-free survival benefit was demonstrated between both groups (11.1 vs 13.1 months, respectively, p=0.43). In contrast, in a post-hoc subgroup analysis, patients with a PCI between 11 and 15 had longer OS in the CRS + HIPEC group than in the CRS alone group (41.6 vs. 32.7 months, p=0.02). The trial analyzed safety outcomes with a similar 30-day MM between groups. However, at 60 days, MM occurred more frequently in the CRS plus HIPEC group compared to the CRS group (respectively, 26% vs 15%, p=0.035). These results were in accordance with those previously published in the literature [7]. The most important intra-abdominal complications were digestive fistulae and abscesses. Although the frequency of extra-abdominal complications was comparable between groups, hemorrhagic complications were higher in HIPEC group, probably related to the use of oxaliplatin drug.

This RCT was the first trial to investigate the specific role of HIPEC in patients with PM of colorectal origin undergoing CRS. Nevertheless, PRODIGE 7 did not demonstrate the overall survival benefit of oxaliplatin based-HIPEC following CRS, thus challenging the clinical practice.

#### HIPEC in patients with colon cancer at high risk of peritoneal metastasis (prophylactic indication)

Ten RCTs were identified evaluating the prophylactic role of HIPEC in 1,983 randomized patients with colon cancer at high risk of PM. Six trials have already been analyzed in the previous review by Eveno et al. [8]. Four new trials are presented in Table 2.

The French PROPHYLOCHIP (or PRODIGE 15) trial [11] investigated a potential favorable effect of a second surgical look combined with HIPEC on disease-free survival (DFS) vs. surveillance in patients at high risk of developing colorectal PM. Accrual patient was 130 but 150 patients were included. Eligible patients had histological proven primary colorectal cancer with synchronous and localized PM, or resected ovarian metastases or a perforated tumor. Moreover, all patients received adjuvant chemotherapy for six months. In absence of disease recurrence on abdominal CT-scan, patients were randomly assigned to standard surveillance only vs. a second-look surgery with oxaliplatin based-HIPEC. After a median follow-up of 50.8 months, 3-year DFS did not differ between second-look surgery group and surveillance group (respectively, 44% vs 53%, p=0.82). Recurrence was located in order of frequency in peritoneum, liver and nodes. Three-year peritoneal recurrence-free survival (RFS) was similar in both groups (respectively, 59% vs 61%). However, grade III/IV complications in the second-look surgery were 41% and the most common complications were intra-abdominal complications (17%) and hematological toxicity (18%).

COLOPEC is an open-label, Dutch randomized multicenter trial [12] and aimed to analyze the peritoneal-free survival benefit of adjuvant HIPEC after curative resection of T4N0-2M0 stage or perforated colon cancer. 204 patients (with a planned accrual patient of 176) were randomized to adjuvant oxaliplatin based-HIPEC or standard treatment. Randomization was carried out before curative surgery of the primary tumor or after histological confirmation (T4 stage or perforation). HIPEC was performed simultaneously or 5–8 weeks after surgery. In both groups, all patients were treated with systemic chemotherapy within 12 weeks after curative resection of the primary tumor. In absence of radiological peritoneal recurrence during surveillance, diagnostic laparoscopy was performed at 18 months. No difference in peritoneal-free survival at 18 months was demonstrated: 80.9% for the HIPEC group compared to 76.2% for the control group (p=0.28). PM was diagnosed in 19% of patients in the HIPEC group and 11% of whom were diagnosed at 18-month laparoscopy (vs. 23% and 30%, respectively, in the control group). DFS and OS were comparable between groups (respectively, p=0.99 and p=0.82).

In Italy, a further RCT (NCT03914820) called CHECK study is currently recruiting 330 patients with colon cancer at high risk of developing PM (stage T4, perforated tumor, ovarian metastases or limited peritoneal disease). Exclusion criterion is presence of distant metastases. This phase III trial randomizes patients into adjuvant mitomycin based-HIPEC or not during surgery, provided that a complete R0 resection was achieved. The primary endpoint is the local RFS defined as the time between randomization and recurrence of PM or death for any cause. Secondary endpoints are DFS, OS, morbidity and mortality rate at 30 and 90-day from surgery.

### Gastric cancer

Table 3 summarizes RCTs evaluating CRS and HIPEC in gastric cancer. Sixteen trials were identified with a planned total of 3,041 randomized patients. Most of them are ongoing and related to the prophylactic role of HIPEC in the prevention of PM of gastric origin.

**Table 3:** Prospective randomized trials evaluating CRS and HIPEC in gastric cancer.

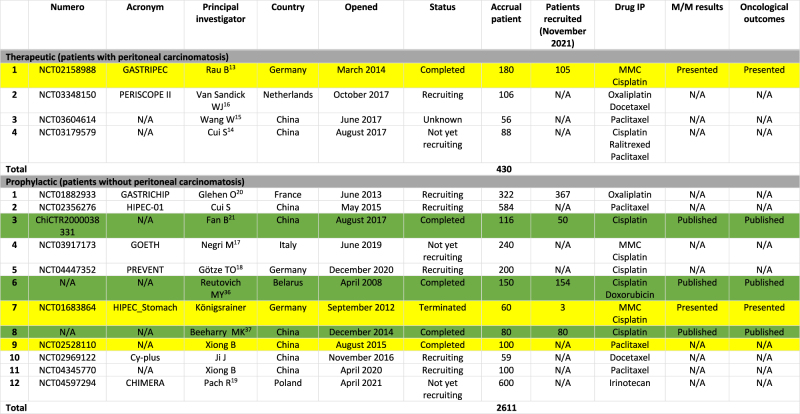

N/A, not available at date (November 2021); MMC, mitomycin. Yellow: trial completed. Green: results published.

#### Therapeutic indication

Four trials analyzed the role of HIPEC in patients with synchronous PM of gastric origin. RCTs are ongoing with a total accrual patient of 430.

The GASTRIPEC trial has already been presented in the previous review [8]. Despite a low recruitment with only 105 patients enrolled for 180 scheduled, the results were recently presented in ESMO [13] and ESSO 2021. A large amount of drop-out (50%) was observed between the first laparoscopy (after IV chemotherapy) and the laparoscopy for CRS leading to a small number of patients in CRS alone group (n=22) compared to CRS + HIPEC (n=28). While median OS did not differ between CRS alone group and CRS + HIPEC group (p=0.16), PFS and other distant metastasis free survival were significantly improved in CRS + HIPEC group compared to CRS alone group (7.1 months vs. 3.5 months, p=0.04 and 10.2 months vs. 9.2 months, p=0.02, respectively).

Two Chinese trials (NCT03179579 and NCT03604614) opened in 2017 [14, 15]. The first one [14] aimed to evaluate median 3-year OS in patients with PM of gastric cancer treated either with CRS and HIPEC or with CRS alone. PCI score of patients had to be less than 20. HIPEC was performed with paclitaxel, cisplatin and raltitrexed. The accrual patient was 88. The current recruitment status is unknown with a last update in 2017. In the second one [15] the experimental and control group were similar but the drug used for HIPEC was Oxaliplatin. Primary outcome measure was PFS. Safety results were identified as secondary endpoints. The recruitment status is unknown and the last update was in 2018.

The Dutch PERISCOPE II study [16] is a controlled two-arm multicenter randomized study comparing CRS and HIPEC vs. palliative systemic chemotherapy. Patients with T3-T4 gastric tumor, limited PM (PCI<7) and/or positive cytology without extraperitoneal metastases are eligible for inclusion. All patients receive systemic chemotherapy prior to inclusion and all regimens are acceptable. In the absence of disease progression, patients are included and then randomized. In the experimental group, a gastrectomy with D2 lymphadenectomy is performed. HIPEC procedure is using an open abdominal technique with oxaliplatin during 30 min followed by Docetaxel for 90 min. Adjuvant treatment is not detailed in the protocol. Primary endpoint is 5-year OS. PFS, toxicity, costs and health benefits are also evaluated 5 years postoperatively. One hundred and six patients have been enrolled for an accrual patient of 182. Estimated study completion date is October 2022.

#### Prophylactic indication

Since the review published in 2016 [8] new promising trials have emerged and are mostly recruiting.

The GOETH study [17] led by Mario Negri was opened in June 2019. The aim is to analyze the efficacy of mitomycin and cisplatin-HIPEC combined with CRS in patients with gastric cancer at high risk of developing PM (T3-T4N0-N+ stage, perforated tumor or cytology positive). Patients with gastroesophageal junction cancer, distant metastases or with synchronous PM are excluded. Randomization is performed intraoperatively if complete resection can be reached. Adjuvant chemotherapy has to be administrated in both groups. The primary objective of the study is 3-year DFS. Secondary end points are 3-year OS, 3-year local RFS, morbidity and mortality.

The Dutch PREVENT trial [18] aims to compare PFS/DFS in patients with locally advanced adenocarcinoma of the stomach (T3-T4, any N, M0) but also gastroesophageal junction (type II/III) with exclusion of distant metastases. After receiving neoadjuvant FLOT-chemotherapy, the experimental group undergoes surgery and HIPEC. In the control group, surgery alone is performed. Curative resection (gastrectomy or transhiatal extended gastrectomy) is planned within 4–6 weeks and drug administered intraperitoneally is cisplatin. All patients receive adjuvant FLOT-chemotherapy (docetaxel, oxaliplatin, leucovorin and 5-fluorouracil) from 6 to 12 weeks maximum after surgery. The number of accrual patients is 200 and the trial is recruiting.

The CHIMERA trial [19], was recently opened in April 2021 in Poland and submitted a similar protocol. CHIMERA is a randomized, multicenter clinical trial with a large accrual patient of 600. Patients with gastric cancer at high risk of developing PM are enrolled. After receiving 4 cycles of FLOT4 chemotherapy, patients are randomly allocated to receive either HIPEC with irinotecan plus surgery or surgery alone, followed by adjuvant chemotherapy with FLOT4 in both groups. Primary outcome criterion is peritoneal recurrence at 6 months postoperative. Patients will be followed for 5 years or until death. Survival results are not expected until 2026.

The most awaited and advanced study is the French GASTRICHIP study conducted by Olivier Glehen [20]. This prospective, randomized, multicenter phase III trial included 367 out of 322 accrual patients. Inclusion criteria were patients with histologically proven resectable gastric adenocarcinoma for which a curative gastrectomy was scheduled, with invasion into the serosa and/or lymph node metastasis and/or positive peritoneal cytology and/or perforated gastric adenocarcinoma and/or Siewert III adenocarcinoma of the cardia for which a gastrectomy by exclusive abdominal laparotomy is scheduled. Patients were treated with a curative gastrectomy with or without Oxaliplatin-based HIPEC. The main objective of the study was 5-year OS. The study is closed to inclusion and the results are expected.

Finally, results of a Chinese study were recently published in 2021 [21]. This prospective phase II study analyzed the survival benefit and toxicity of prophylactic HIPEC in the same type of population than in other studies. The trial was stopped after inclusion of 50 patients (with an initial need of 116 patients). After radical gastrectomy, patients were randomized (2:1) with or without HIPEC. HIPEC was administered with cisplatin drug during 30 min. All patients received adjuvant chemotherapy with SOX regime. Survival results did not show a survival advantage for the experimental group. After a median follow-up of 37 months, 3-year RFS rate was 84.2% in the experimental group compared with 88.2% in the control group (p=0.986). Overall, 3-year survival of patients with or without HIPEC was 87.9% and 100%, respectively (p=0.142). Safety results demonstrated however that HIPEC was well tolerated with no significant difference in postoperative complications rate (p>0.05).

### Ovarian cancer

Published and ongoing RCTs evaluating the effect of HIPEC in ovarian cancer are summarized in Table 4.

**Table 4:** Prospective randomized trials evaluating CRS and HIPEC in ovarian cancer.

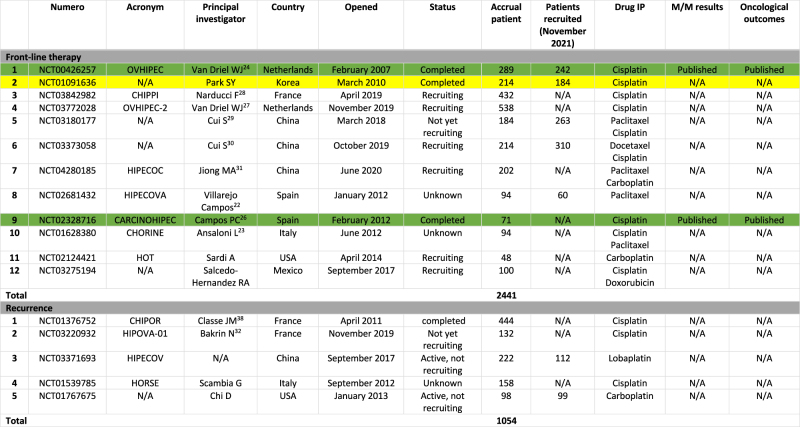

N/A, not available at date (November 2021). Yellow: trial completed. Green: results published.

#### Front-line therapy

Currently, there are still no published trials on the impact of HIPEC in front-line treatment of ovarian cancer for primary surgery. Twelve RCTs have been initiated with a planned total of 2,441 randomized patients: six trials are ongoing; one is completed and results have not yet been presented. The statuses of three studies previously described by Eveno et al. are unknown at this time [22, 23].

The multicentric Dutch OVHIPEC trial [24], published in 2018, analyzed 245 patients with stage III epithelian ovarian, fallopian tube, or peritoneal cancer. Patients eligible for secondary debulking were women with an incomplete primary cytoreduction (one or more residual tumors >1 cm diameter were present) or those who were unable to have cytoreduction because of extensive abdominal disease. Randomization was performed intraoperatively if complete cytoreductive (CC0 or CC1) was achieved. After undergoing interval surgery, patients were randomized with or without cisplatin-HIPEC. Adjuvant chemotherapy was mandatory in both groups with three cycles of carboplatin and paclitaxel. Recurrence survival was defined by increase of CA125 level and Response Evaluation Criteria in Solid Tumors (RECIST) in CT scan, as recommended by Gynecological Cancer InterGroup (GCIG) [25]. At a median follow-up of 4.7 years, the median RFS in the surgery group was 10.7 months compared to 14.2 months in the surgery-HIPEC group. The median OS was higher in patients who underwent CRS/HIPEC than patients who underwent CRS alone (45.7 vs 33.9 months). Adverse events of grade 3 or 4 were similar in both group (respectively, 25 and 27%, p=0.76). The most grade III/IV complications were abdominal pain, infection and ileus. This study is one of the first to demonstrate the survival benefit of combining HIPEC to secondary CRS in patients with ovarian cancer.

Campos et al. published recently a randomized, prospective phase 3 trial for patients with PM from epithelial ovarian or fallopian tube cancer [26]. All patients received at least three cycles of systemic neoadjuvant chemotherapy with carboplatin and paclitaxel. Surgery was performed 4 weeks after systemic chemotherapy and 71 patients were randomized to undergo CRS alone (36 patients in the control group) or CRS combined with HIPEC (35 patients in the experimental group). The primary outcome was DFS, defined as the time from surgery to disease recurrence or death and based on the serologic determination of CA 125 and radiological tests. With a median follow-up of 32 months, DFS and OS were better in the experimental group (18 vs 12 months and 52 vs 45 months, respectively). Morbidity and mortality did not differ in both groups (p>0.05).

The OVHIPEC-2 study [27], which opened in November 2019 by Willemien Van Driel, is a multicenter phase III trial designed to prove the benefit of adjoining HIPEC with CRS in ovarian cancer patients with acceptable morbidity. This large trial with an accrual patient of 538 includes women with FIGO III epithelian ovarian cancer and eligible for primary CRS with no residual disease (CC0) or residual disease up to 2.5 mm (CC-1). Eligible patients are randomized into primary CRS with or without cisplatin-based HIPEC. Six cycles of carboplatin–paclitaxel as adjuvant chemotherapy with or without PARP inhibition or bevacizumab according to international recommendations is administrated in both groups. With a follow-up of 5 years, overall survival as primary criteria is analyzed by CA125 and CT-scan and defined as the time from randomization to the date of death from any cause. Secondary endpoints are RFS, treatment-related toxicity and morbidity.

The French CHIPPI trial [28], another large trial, which opened the same year in France, also investigates the addition of HIPEC to primary or interval surgery in ovarian cancer patients. Accrual patients were 432. After surgery, patients were randomly assigned to receive HIPEC or not. Interval debulking surgery is performed after an interval of 3–5 weekends (4–6 weeks if bevacizumab is added to chemotherapy). HIPEC were administered for 90 min using cisplatin. DFS is assessed up to 5 years. Secondary objectives of the study included OS, morbidity, mortality and quality of life. The primary completion date is estimated for 2023.

Three trials are ongoing in China and consecutively opened in 2018, 2019 and 2020 [29], [30], [31]. Three trials analyze oncologic outcomes of adjuvant HIPEC following CRS in patients with ovarian cancer. CRS is performed if Fagotti score is less than 6 during laparoscopic exploration. Drugs and protocols of HIPEC differ between studies.

#### Recurrence

Five trials are designed in recurrent ovarian cancer with a planned total of 1,054 randomized patients.

The CHIPOR study, opened in April 2011, is a phase III randomized study and evaluate the impact on OS of adjunction of HIPEC to CRS in patients with first relapsed ovarian and platinum-sensitive ovarian cancer. Over May 2021, over 400 patients have been included and results are awaited.

The HIPOVA-01 study, a French trial led by Naoual Bakrin, which is currently not yet open, aims to evaluate the effectiveness of adding surgery and HIPEC to the systemic treatment of platinium-resistant recurrence [32]. After receiving chemotherapy and bevacizumab, patients will be randomly assigned to the cytoreductive surgery and cisplatin-HIPEC followed by adjuvant systemic therapy or chemotherapy with bevacizumab alone (called “Aurelia arm”). Oncological outcomes will be analyzed over a 36-month follow-up period. The trial is not yet recruiting with an accrual patient of 132.

### Other histologies

There is a single RCT of patients with resectable pancreatic adenocarcinoma [33]. This phase II–III study aims to evaluate the benefit of HIPEC combining with CRS, their hypothesis being that this combined treatment would decrease the tumor progression of pancreatic cancer, thus improving survival and decreasing the potential recurrence of the disease.

## Discussion

Numerous published, ongoing or planned RCT are analyzing the efficacy and safety of CRS and HIPEC vs. other strategies in therapy and prevention of PM. To date, 46 studies randomized 9,063 patients with PM: 13 RCTs in colorectal cancer (3 in therapeutic indication and 10 for prevention), 16 RCTs in gastric cancer (4 for therapy and 12 for prevention) and 17 RCTs in ovarian cancer (12 in front-line therapy and 7 for recurrence). There is only one RCT in PM of pancreatic cancer and none in primary peritoneal malignancies (such as MPM and PMP). Nevertheless, few studies have been published in those different indications (5 in colorectal cancer, 3 in gastric cancer and 1 in ovarian cancer).

In colorectal peritoneal carcinomatosis, the most awaited and recently published trial, PRODIGE-7, was the first to evaluate the addition of HIPEC to CRS [10]. Quenet et al. demonstrated no difference in overall survival between CRS and HIPEC compared to CRS alone (41.2 vs. 41.7 months, respectively). However, this study confirmed the important central role of complete CRS in the surgical management of peritoneal metastases of colorectal origin with a median overall survival of 41 months compared to palliative treatment [34] or systemic chemotherapy alone [5]. This study questioned the contribution of HIPEC with oxaliplatin for patients with colorectal cancer. This study as some methodological flaws that should be underlined: (i) sixteen patients of the CRS alone group received HIPEC, achieving a 12% rate of crossover; (ii) randomization was not stratified on PCI, leading to a higher rate of PCI>15 in CRS + HIPEC group of 30% vs. 20% in CRS alone; (iii) more MM was found in CRS + HIPEC group with a 30-days MM of 42% vs 32% and a 60-days MM of 26% vs. 15%, with an 4 fold-increase rate of hemorrhagic complication in CRS + HIPEC group (9.8% vs 2.3%) attributed to the toxicity of oxaliplatin-HIPEC regimen [highly heated (43°) and dosed (460 mg/m^2^ for the open technique and 360 mg/m^2^ for the closed technique) with a short time-exposure (30 min)].

Furthers reflection is needed to conduct RTCs with better selection of patients (lower PCI), no crossover between groups of randomization and decreased morbidity expected with other types of drugs (mitomycin C, cisplatin) and lower PCI.

Unlike therapeutic studies, many studies ongoing evaluated prophylactic HIPEC in preventing PM of colorectal origin. Some trials have already been published such as PROPHYLOCHIP [11] and COLOPEC trials [12], and did not demonstrate a survival advantage in oxaliplatin based-HIPEC compared to standard treatment in patients at high risk of developing PM. Reflection is made to better select criteria of inclusion (pT4, pN2, mucinous tumor, ovarian or limited PM) and the best timing of surgery and HIPEC in patients at high risk of peritoneal recurrence.

Beside the probable inefficacy of the French-oxaliplatin-based HIPEC regimen, PROPHYLOCHIP and COLOPEC trials raised some comments: in the first one, (i) 10% (8 patients on 75) of the experimental group with HIPEC had a crossover and did not received any HIPEC; (ii) peritoneal recurrence rate in the 2 groups was very different with 52% in the HIPEC group and 34% in the surveillance group, raising the problem of not centralized monitoring of the CT-scan for the primary objective; (iii) in the surveillance group, 16 of the 25 patients (64%) with peritoneal recurrence had CRS + HIPEC; (iv) the risk of peritoneal recurrence after perforated tumors was lower than expected (16% PM in 64 patients), questioning the criteria of selection of patients with high risk of recurrence in the peritoneum.

In the second one, 9 patients of the HIPEC group (9%) had already PM during laparoscopy at 5–8 months of the laparoscopic colectomy, questioning the poor initial assessment in nonspecialized centers or deleterious effect of laparoscopy.

Efforts are being made to better select patients and timing of prophylactic exploration and treatment, as well as the best HIPEC drug to improve prognosis of patients with high risk of recurrence under PM.

The encouraging Italian CHECK study is not expected before 2025 to evaluate the potential advantage of mitomycin-HIPEC combined to CRS [35].

In gastric cancer, no randomized studies have been published in the therapeutic indication. Nevertheless, only four RCTs are currently in progress. The most advanced trial is PERISCOPE II [16] is expected to demonstrate the positive effect of CRS and HIPEC on survival of patients with limited peritoneal metastases (PCI<7). In prophylactic situations with patients at high risk of developing PM (T3-T4, N+), more than ten studies were identified. Two published trials are in favor of HIPEC with cisplatin improving DFS [36, 37]. GASTRIPEC trials showed promising results on PFS and on MM with CRS + MMC and cisplatin-based HIPEC [13]. However, major dropout rate of 50% and limited number of randomized patients make the results difficult to be interpreted. These encouraging results should be supported by the large Dutch PREVENT trial (NCT04447352) recruiting over 600 patients, the data of GOETH study (NCT03917173) or even Poland CHIMERA (NCT04597294). As for GASTRICHIP study [20], recruiting is completed and results are awaited.

In the interval situation, Van Driel et al. published the OVHIPEC trial [24], the first to demonstrate the major role of complete CRS combining with cisplatin-HIPEC in interval surgery, showing major improvement of median overall survival (12 months) and RFS (4 months) compared to surgery alone. Promising randomized trials are currently recruiting in front line situation with different protocols of cisplatin based-HIPEC compared to CRS alone. The large Dutch OVHIPEC-2 [27] and French CHIPPI studies [28] are expected to prove the survival benefit of adding HIPEC to CRS in primary setting with an accrual patients of 538 and 432, respectively. Two studies will define the therapeutic management in women with recurrent ovarian cancer in platinum-resistant [HIPOVA-01 [32]] and platinum-sensitive recurrence [CHIPOR [38]].

In conclusion, numerous studies have confirmed the positive effect on overall survival of cytoreduction surgery in the management of PM. In view of the different randomized studies analyzed in our review, the role of HIPEC is still debated in some indications but tends to play a major role in highly selected patients with PM. Promising studies are currently underway to corroborate or not these hypotheses.
